# Editorial: Forces in biology - Cell and developmental mechanobiology and its implications in disease - Volume II

**DOI:** 10.3389/fcell.2022.1082857

**Published:** 2022-12-01

**Authors:** Selwin K. Wu, Guillermo A. Gomez, Samantha Stehbens, Bipul R. Acharya, Aparna Ratheesh, Rashmi Priya, Anne Lagendijk, Alexander Bershadsky

**Affiliations:** ^1^ Mechanobiology Institute, National University of Singapore, Singapore, Singapore; ^2^ Department of Biological Sciences, National University of Singapore, Singapore, Singapore; ^3^ Centre for Cancer Biology, SA Pathology and University of South Australia, Adelaide, SA, Australia; ^4^ Cell and Developmental Biology Division, Institute for Molecular Bioscience, The University of Queensland, Brisbane, QLD, Australia; ^5^ Wellcome Centre for Cell-Matrix Research, Faculty of Biology, Medicine & Health, Manchester Academic Health Science Centre, University of Manchester, Manchester, United Kingdom; ^6^ Warwick Medical School, University of Warwick, Coventry, United Kingdom; ^7^ Francis Crick Institute, London, United Kingdom; ^8^ School of Biomedical Sciences, The University of Queensland, Brisbane, QLD, Australia; ^9^ Department of Molecular Cell Biology, Weizmann Institute of Science, Rehovot, Israel

**Keywords:** forces, brain, cancer, bones and cartilage, immunology

Cellular biomechanics underlie a diversity of cellular processes and behaviours, simultaneously being a fundamental determinant of tissue patterning. Core cellular activities, from differentiation and proliferation to migration and apoptosis, are influenced by the cell’s mechanical properties and the ability to discern mechanical signals from their environment. The intrinsic cell machinery internalizes exogenous physical signals from their environment, and a high-precision regulation from a molecular to multicellular level ensures each cell interpret such signals and meets its designated fate. Thus, cells within tissues constantly acquire sensory inputs from their neighbours and the extracellular matrix—detecting, transducing and processing mechanochemical signals to modulate processes and coordinate responses. In this issue, we highlight recent developments in three areas of cell biomechanics in these chapters: 1) Cell and Developmental Biology, 2) Cancer and 3) Bones & Connective Tissues.

In the first chapter, Alvarez and Smutny reviewed the recent advancements on how actomyosin-contractile forces affect tissue morphogenesis and regulate cell fate in mouse and zebrafish embryonic development. Next, Procès et al. developed a comprehensive review of the heterogenous mechanobiology of the brain, from a molecular to organ level, to help engineer early interventional therapies or treatments for traumatic brain injury, neurodegenerative diseases, and glioblastoma.

As the complexity of our understanding of mechanobiology grows, theoretical modelling can help interweave existing experimental data and consolidate various parameters into a singular, quantitative system. These computational models can help to form new hypotheses or to unify concepts. Khataee et al. put forward a general model based on the Cellular Potts Model to encapsulate and analyze the interplay between cellular mechanical properties and dynamical transitions in epithelial cell shapes and structures. Interestingly, cell extrusion promotes monolayer-to-multilayer transition based on the mechanical properties of cells and the orientation of cell division.

While mechanochemical signalling pathways can control both cellular dynamics (at a short timescale) and gene expression (at longer time scales), their co-regulation is critical for the self-organization of cells into tissues. Afzal et al. studied how the interaction of placenta and the endometrium eventually results in the deep invasion of placental extravillous trophoblasts into the maternal stroma. They demonstrated that paracrine HB-EGF signalling reduces activated decidual fibroblasts’ enhanced contractility and energetic state by rebalancing the SRF-MRTF-TCF transcriptional axis.

Further, misregulation of the aforementioned mechanobiological mechanisms can lead to pathological consequences such as cancer and suppressed immunity. In Chapter 2, we focus on cancer mechanobiology. Dobrokhotov et al. found that actomyosin activity is impaired in cutaneous squamous cell carcinoma (cSCC). External application of tensile loads to adherens junctions by sustained mechanical stretch attenuates the proliferation of cSCC cells. Force-dependent activation of actomyosin in cSCC cells also inhibits their proliferation in a cell-cell contact-dependent manner. Taken together, the malignancy of cSCC cells may be reduced by applying tensile loads to adherens junctions.

Besides the adherens junctions, cancer progression also involves remodelling the extracellular matrix (ECM). Vahala and Choi review current platforms and biomaterials engineered to mimic the micro and nano-properties of the tumour microenvironment, and subsequent understanding of mechanically regulated pathways in cancer. In essence, cancer cells morphologically adapt to survive in altered environments. Thus, changing tumour ECM properties, including stiffness and ligand chemistry and spacing, are factors that should be considered and incorporated when designing future tools.

Next, how contractile forces regulate non-adherent natural killer (NK) cells during cancer surveillance was addressed by Wong et al.

They found that lung cancer cells can provoke NK cells and enhance their actomyosin-mediated contractility as a potential early phase activation mechanism. This action shuttles Eomes, an evolutionarily conserved NK cell transcription factor, into the nucleus. NK cells responded to the presumed immunosuppressive TGFβ in the NK-lung cancer coculture medium to sustain its intracellular contractility through myosin light chain phosphorylation, thereby promoting the nuclear localization of Eomes, which likely responds downstream to mechanical stimuli for increased NK cytotoxicity.

In the final chapter, we focus on the study of bones and connective tissues, which lies precisely at the interface of biomechanics and mechanobiology. The chapter commences with Choi et al. reviewing the current knowledge of the osteocyte’s role in maintaining bone health and the key regulatory pathways of these mechanosensitive cells. Subsequently, they highlighted the therapeutic opportunities offered by existing treatments and the potential for targeting osteocyte-directed signalling.

Delving into the cartilage, Boos et al. reviewed the different cartilages and chondrocyte mechanosensing types, then moved on to the multiscale strain transfer through cartilage tissue the involvement of individual ECM components before finally outlining insights to understand multiscale strain transfer in cartilage further. Essentially, the heterogeneity in the spatial variation of ECM molecules leads to a non-uniform, depth-dependent strain transfer and alters the magnitude of forces sensed by cells in articular cartilage and fibrocartilage, influencing chondrocyte metabolism and biochemical response.

We end the issue with a review of Ehlers-Danlos Syndromes (EDSs), a group of connective tissue disorders characterized by skin stretchability, joint hypermobility and instability. Though EDS patients typically exhibit lowered elasticity, recent evidence suggests that comorbidities of EDS could also be associated with reduced tissue stiffness. Royer and Han discussed the potential mechanobiological pathways involved in the two most popular types of EDS: classical and hypermobile, and their respective, associated comorbidities: mast cell activation syndrome and impaired wound healing—finding that altered mechanosensitive proteins and the lack of collagen V to be main contributors respectively.

Extending the traditions of our first issue (Forces in Biology) (Wu et al., 2020), we hope that this issue will also be of exceptional interest to students and researchers studying molecular and cellular mechanisms in development and diseases; To understand and appreciate their complex, yet cohesive inner workings, and ideally inspire better designs in therapeutics and diagnostics.
